# Correlation Study between Civilian SARS-CoV-2 Wastewater Concentration and COVID-19 Cases among U.S. Military Health System Beneficiaries

**DOI:** 10.4269/ajtmh.26-0253

**Published:** 2026-11-12

**Authors:** Sneha P. Cherukuri, Mark L. Bova, Shaylee P. Mehta, Emily R. Tholen, Christian T. Bautista

**Affiliations:** Integrated Biosurveillance Branch, Defense Health Agency – Public Health Directorate, Armed Forces Health Surveillance Division, Silver Spring, Maryland, USA

## Abstract

Wastewater-based epidemiology has emerged as a new near real-time tool for population-wide COVID-19 surveillance. This study examined the statistical correlation between county-level civilian SARS-CoV-2 wastewater concentration and COVID-19 cases among beneficiaries of the U.S. Military Health System (MHS) from April 2024 to April 2025 in the continental United States. The analysis revealed an overall high correlation for both MHS beneficiaries (*rs =* 0.797; 95% CI: 0.626–0.893) and active-duty service members (*rs =* 0.800; 95% CI: 0.633–0.892). Further, an assessment of external factors suggested that correlations were stronger in regions with higher MHS populations. Our findings indicated that civilian SARS-CoV-2 wastewater served as a robust early indicator for COVID-19 trends among MHS beneficiaries and that integrating civilian wastewater data may enhance near real-time Department of War health surveillance monitoring of COVID-19.

## INTRODUCTION

Respiratory infectious diseases remain a leading cause of global morbidity and mortality. Active-duty service members (AD SM) often face an increased risk as a result of overcrowding, limited health care access, and high-stress operations.^1^ These environments can facilitate the rapid transmission of pathogens such as SARS-CoV-2. Therefore, early detection of emerging health threats is critical. However, existing disease surveillance systems are often hindered by diagnostic and reporting lags, typically days or weeks after infection, failing to capture the trajectory of an epidemic in real time.^2^

Because both symptomatic and asymptomatic individuals shed the SARS-CoV-2 virus via stool, wastewater testing has emerged as a tool for monitoring community-level infection trends.^3^ This tool can also provide near real-time data and serve as a leading indicator^4^ to predict trends in key disease metrics, such as cases and hospitalizations, from days to weeks in advance.^5^^,^^6^ Furthermore, wastewater-based epidemiology (WBE) can overcome the limitations of traditional disease surveillance systems, because it remains independent of health care–seeking behaviors and testing availability. Despite the growing use of WBE, research on its application to health information from the U.S. Military Health System (MHS) beneficiaries remains limited. During fiscal year 2024, there were 9.4 million MHS beneficiaries composed of AD SMs, military retirees, and their families,^7^ many of whom live in communities overlapping with sewer sheds in nearby cities.^8^ Establishing a strong correlation between wastewater and MHS cases and identifying an increasing wastewater signal could provide extra time for decision-makers within the U.S. Department of War (DoW) enterprise to mobilize resources and enhance protection measures across health care infrastructure, therefore reducing the burden of disease.

This study integrated weekly civilian (CIV) wastewater data from the U.S. Centers for Disease Control and Prevention’s National Wastewater Surveillance System (NWSS)^9^ with health records from the U.S. DoW to assess the correlation between SARS-CoV-2 wastewater concentration and the number of COVID-19 cases in the MHS population from 27 April 2024 (epidemiological week [EW] 17) through 26 April 2025 (EW 17).

## MATERIALS AND METHODS

For this study, the Armed Forces Health Surveillance Division’s Integrated Biosurveillance Branch (IB), which is responsible for the monitoring and reporting of health threats of military interest worldwide, extracted COVID-19 case data for MHS beneficiaries from the Defense Medical Surveillance System (DMSS) and the DoW’s Electronic Surveillance System for the Early Notification of Community-based Epidemics (ESSENCE) from April 27, 2024 through April 26, 2025. A COVID-19 case was defined as a positive laboratory test for SARS-CoV-2. The EWs of the specimen collection dates were used for analysis.

For surveillance purposes, these data, reported by Military Treatment Facilities (MTFs), are organized by the IB branch into surveillance regions within the continental United States (CONUS) by mapping neighboring MTFs. A catchment area for civilian data surrounding each surveillance region was created by including all counties located within a 30-mile radius of each MTF within the surveillance region. Civilian SARS-CoV-2 wastewater concentrations by county were collected from the NWSS, covering 49 U.S. states (excluding North Dakota).^9^ The SARS-CoV-2 wastewater concentration data are reported by sample collection date and in units of gene copies per liter at each wastewater treatment plant (site). Standardized county Federal Information Processing Standards (FIPS) code information was extracted for each reporting site and then aggregated to IB surveillance region by calculating a weekly median value.

We restricted the analysis to IB surveillance regions with complete civilian wastewater data (53 reporting weeks) and at least one MHS COVID-19 case during the study period. Statistical correlations were quantified using Spearman’s rank correlation coefficients (*rs*), with 95% CIs derived via nonparametric bootstrapping. Correlation strength was categorized as negligible (*rs* <0.3), low (0.3 ≤ *rs* <0.5), moderate (0.5 ≤ *rs* <0.7), or high (*rs* ≥0.7). These thresholds are based on a general rule of thumb defined by Hinkle et al.^10^ and prior COVID-19 wastewater correlation studies using the Spearman rank measures.^4^^,^^11^^,^^12^ Each region was categorized by U.S. Census division and the following MHS population categories: 0–<10,000; 10,000–<18,000; 18,000–<45,000; 45,000–<100,000; and 100,000–290,000.

All statistical analyses were conducted using R v. 4.4.2 (R Project for Statistical Computing). The map in Figure 2 was created using ArcGIS Pro v. 3.3.0.

## RESULTS

During the study period, there were 61 IB surveillance regions in the CONUS. Of these, 43 were selected based on the completeness of their wastewater data and 36 (59%) had at least one COVID-19 case over the study period. Of the 36 surveillance regions, eight were in the 0–<10,000 population category; six in the 10,000–<18,000 category; 10 in the 18,000–<45,000 category; 7 in the 45,000–<100,000 category; and 5 in the 100,000–290,000 category. The five largest surveillance regions were the National Capital Region, Tidewater, Puget Sound, North Carolina, Central, and Central Texas. Of the selected regions, there were 18 in the South, six in the West, seven in the Midwest, and five in the Northeast. Within this period, 17,908 COVID-19 cases were reported among MHS beneficiaries (9,913 cases in AD SMs).

Our analysis revealed that MHS COVID-19 cases peaked during EWs 31 and 33 of 2024 (ending August 3 and 17, respectively), matching a peak of SARS-CoV-2 wastewater concentration during EW 31 of 2024 (August 3; Figure 1). A second peak in wastewater concentration occurred during EWs 5 and 6 of 2025 (February 1 to February 8), coinciding with the second peak in MHS COVID-19 cases during the same period (Figure 1). Additionally, the lowest number of MHS COVID-19 cases were recorded during EW 45 and 48 of 2024 (November 9 and November 30, respectively), which was similar to the period of the lowest SARS-CoV-2 viral concentrations during EWs 44 and 47 of 2024 (November 2 and November 23, respectively).

**Figure 1.**
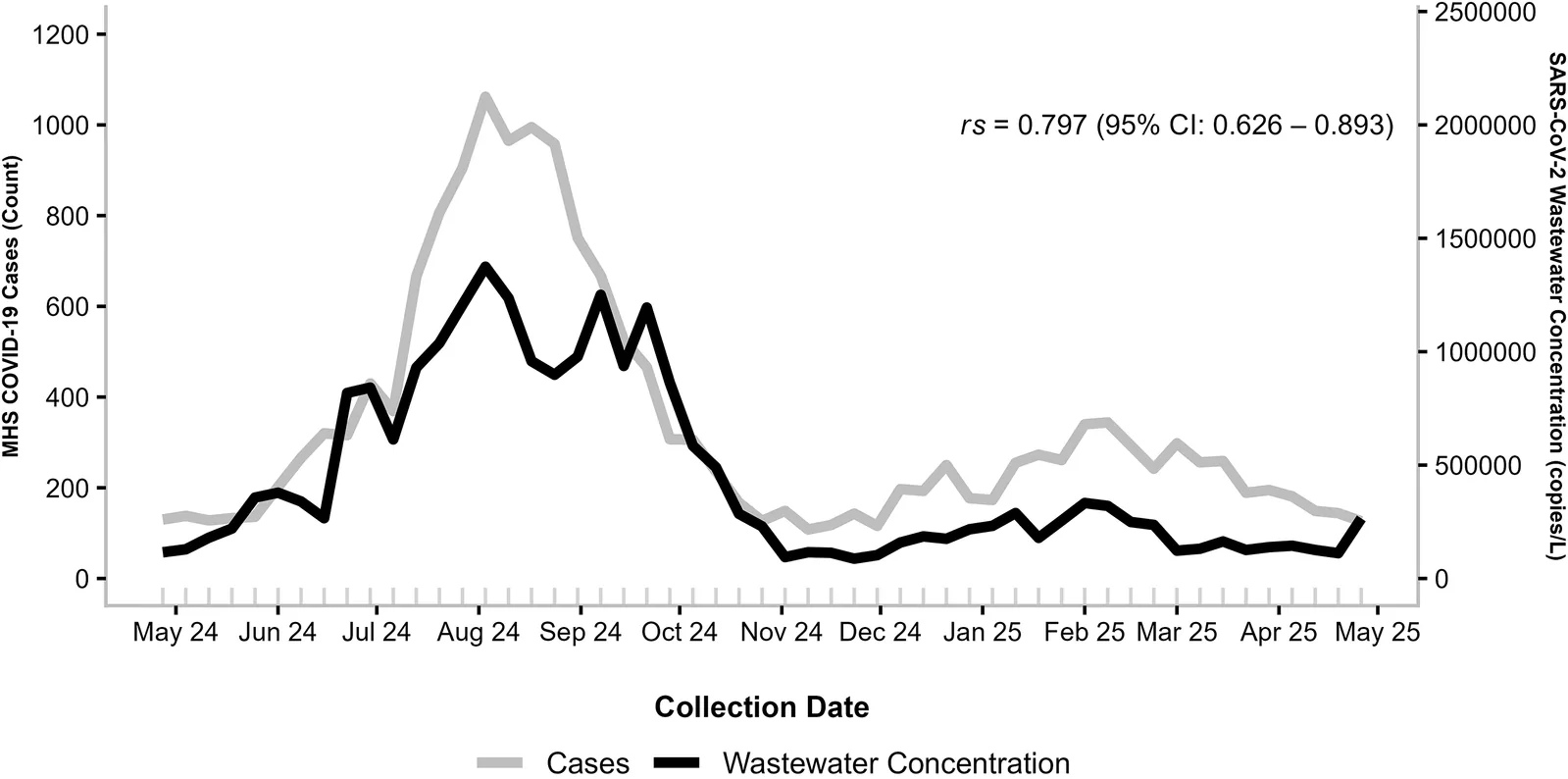
The U.S. Military Health System COVID-19 cases and wastewater concentration in the continental United States, April 2024 to April 2025. A total of 36 Integrated Biosurveillance Branch surveillance regions were included, and a Spearman correlation coefficient was calculated with 95% CIs. Date represents collection date for both SARS-CoV-2 laboratory samples and SARS-CoV-2 wastewater samples.

The Spearman analysis revealed an overall high correlation (*rs =* 0.797; 95% CI: 0.626–0.893) between CIV wastewater concentration and MHS COVID-19 cases. A similarly high correlation was found for only AD SMs (*rs =* 0.800; 95% CI: 0.633–0.892). Our analysis also revealed that, of the 36 selected surveillance regions, 12 had negligible correlations (range *rs = −*0.095–0.287), 8 had low correlations (range *rs =* 0.328–0.462), 12 had moderate correlations (range *rs =* 0.514–0.696), and 4 had high correlations (range *rs =* 0.702–0.839) (Figure 2). Supplemental Table 1 includes census division, population sizes, population categories, and Spearman correlations for each of the 36 surveillance regions. No cases were observed among non-AD SM beneficiaries in the Great Lakes and South Florida regions. Supplemental Table 2 includes a data dictionary of variables that were used in this study and how they were measured or collected.

**Figure 2.**
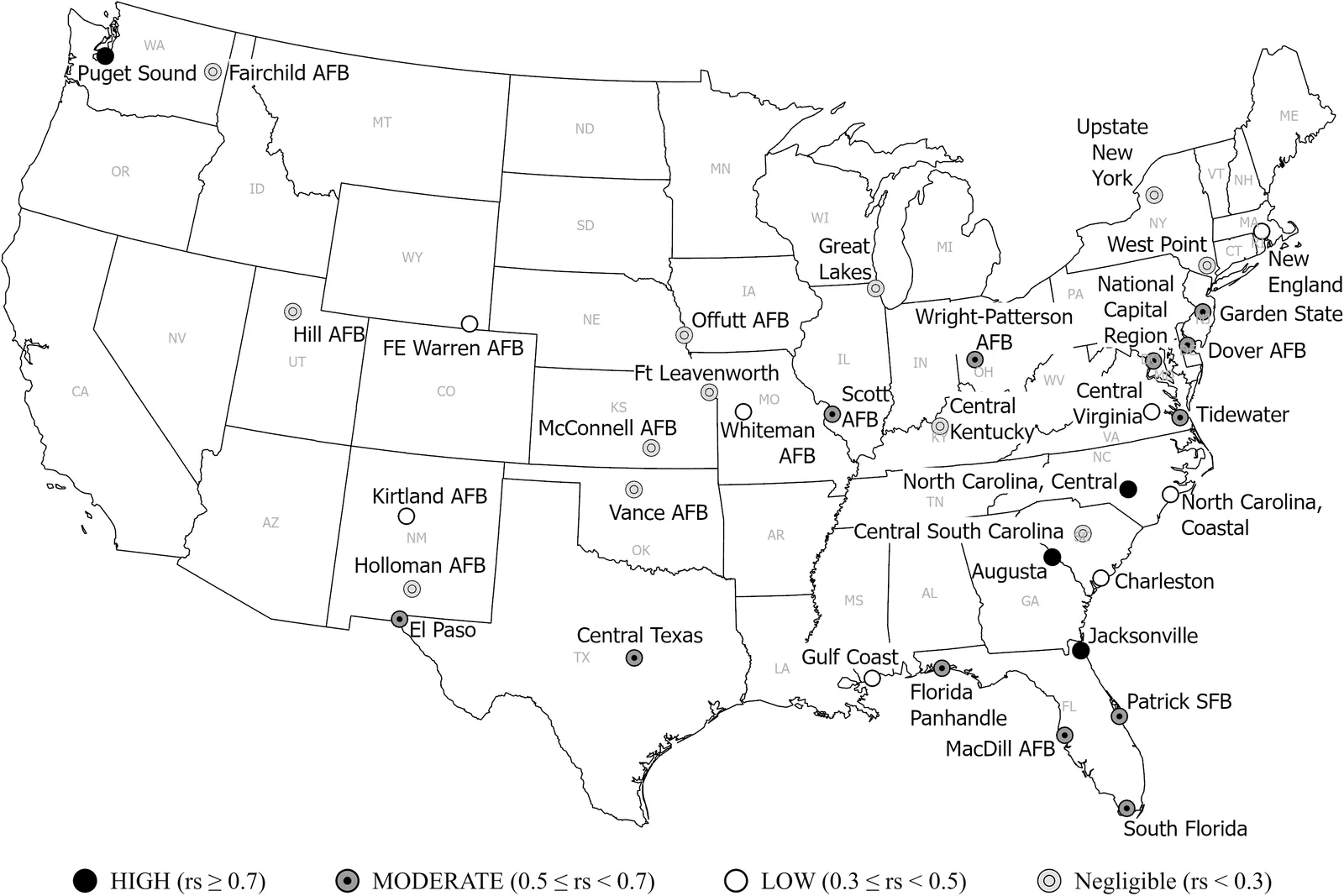
Spearman correlations between the U.S. Military Health System COVID-19 cases and wastewater concentration by Integrated Biosurveillance Branch surveillance region from April 2024–April 2025.

## DISCUSSION

This study identified a high correlation between MHS COVID-19 cases and CIV SARS-CoV-2 wastewater concentrations across the CONUS. During peaks periods in August 2024 and February 2025, wastewater concentrations were highest during the same weeks as MHS COVID-19 cases. However, during periods of low infectivity during November 2024, we observed wastewater concentrations leading COVID-19 cases by 1 week, suggesting that SARS-CoV-2 wastewater concentrations may be a stronger early indicator during nonpeak periods. The overall high correlation and 1-week lead time were consistent with previous WBE papers.^12^ However, the trend observed during peak periods should be interpreted with caution given that wastewater concentrations and cases were aggregated at the weekly level in our analysis. Similar correlation papers have found that wastewater signals lead cases and hospitalizations by 2 to 8 days.^13^^–^^15^ Therefore, a true leading signal measured in days may not be captured with the existing methodology. Correlations were also strongest in the highest MHS population category (100,000–290,000), with the five largest IB surveillance regions (average *rs =* 0.74) making up over 48% of the population in the CONUS, whereas correlations were weaker in the five lowest population regions (average *rs =* 0.347) (Supplemental Table 1), suggesting a potential association between population size and correlation strength. Additionally, no patterns in correlation strength were identified by census division (Supplemental Table 1) or other factors as potential drivers for correlation strength.

From May 2021 to January 2022, the U.S. Air Force conducted a pilot study across 17 Air Force installations and bases to evaluate the economic viability of SARS-CoV-2 wastewater testing. This cost-model analysis concluded that the implementation of SARS-CoV-2 wastewater surveillance is a cost-effective biosurveillance strategy when compared with individual clinical swab testing.^16^ This finding endorses the integration of wastewater monitoring as an additional tool for tracking diseases across U.S. military installations.

This study is subject to at least four limitations. First, due to the absence of a comprehensive DoW SARS-CoV-2 wastewater surveillance program during the study period, civilian NWSS data was used as an early indicator for community viral burden around MTFs. However, the high mobility of the MHS population, including temporary duty assignments, allows individuals to contract infections in other surveillance regions and transmit them to their residential areas. These occurrences may, in turn, weaken statistical correlations across surveillance regions if COVID-19 cases are overrepresented in some surveillance regions and underrepresented in others. Secondly, MHS beneficiaries living in military housing may be serviced by a separate wastewater system not captured within the NWSS. In the current study, it was not possible to discern whether MHS beneficiaries were living in the community or military housing. This circumstance could also have explained differences in correlation strength across the IB surveillance regions. Thirdly, specific geolocations of individual wastewater treatment sites and their sewersheds were not available. Therefore, the spatial distance between sites within the same county could not be assessed, and the use of median wastewater concentrations across sites may mask localized variation in SARS-CoV-2 signal within larger counties. Finally, we did not assess vaccination coverage, which may have influenced infection rates and affected our correlation estimates. Despite these limitations, this study represents a foundational effort to systematically integrate and correlate MHS clinical outcomes with national civilian wastewater surveillance data.

## CONCLUSION

In summary, this study demonstrates that civilian SARS-CoV-2 wastewater concentrations serve as a robust early indicator for COVID-19 trends among MHS beneficiaries in the CONUS, with signals appearing 1 week ahead of cases during nonpeak periods. The overall high correlation found suggests that integrating civilian wastewater data may enhance DoW health surveillance capabilities for COVID-19 in the MHS population. Further research should focus on refining these correlation analyses by expanding surveillance to include other respiratory pathogens, such as influenza—a topic made even more relevant by the recent lifting of influenza vaccine mandates^17^—and conducting similar analyses in strategically significant countries or regions outside the United States.

## Supplemental Materials

10.4269/ajtmh.26-0253 Supplemental Materials
