# Supplementary material for: Correlation Study between Civilian SARS-CoV-2 Wastewater Concentration and COVID-19 Cases among U.S. Military Health System Beneficiaries
Source: Am J Trop Med Hyg. 2026 Nov 12:tpmd260253. Online ahead of print. doi: 10.4269/ajtmh.26-0253 (PMC13770345; doi:10.4269/ajtmh.26-0253)
Supplement: Supplementary file 1 [file tpmd260253.SD1.pdf]

Supplementary Table 1: Spearman Correlations by IB Surveillance Region in the continental U.S.

Surveillance Region	Census Division	MHS Population	Population category	MHS *rs* (95% CI)	AD SM *rs* (95% CI)	Non-AD SM *rs* (95% CI)
**Augusta**	South	45,907	45–100k	0.702 (0.512 – 0.826)	0.600 (0.370 – 0.764)	0.692 (0.500 – 0.813)
**Central Kentucky**	South	15,765	10–18k	0.121 (-0.154 – 0.394)	0.132 (-0.158 – 0.411)	0.162 (-0.137 – 0.437)
**Central South Carolina**	South	50,626	45–100k	0.173 (-0.155 – 0.458)	0.183 (-0.142 – 0.463)	0.155 (-0.193 – 0.431)
**Central Texas**	South	107,452	100–290k	0.691 (0.473 – 0.829)	0.622 (0.376 – 0.779)	0.704 (0.532 – 0.815)
**Central Virginia**	South	23,658	18–45k	0.396 (0.127 – 0.638)	0.380 (0.118 – 0.612)	0.481 (0.205 – 0.703)
**Charleston**	South	43,588	18–45k	0.328 (0.010 – 0.595)	0.273 (-0.045 – 0.551)	0.589 (0.329 – 0.788)
**Dover AFB**	Northeast	8,007	0–10k	0.530 (0.277 – 0.725)	0.513 (0.275 – 0.700)	0.328 (0.039 – 0.587)
**El Paso**	South	95,239	45–100k	0.618 (0.413 – 0.754)	0.423 (0.167 – 0.642)	0.616 (0.442 – 0.740)
**Fairchild AFB**	West	8,612	0–10k	0.001 (-0.244 – 0.252)	0.028 (-0.222 – 0.270)	0.044 (-0.144 – 0.234)
**FE Warren AFB**	West	7,765	0–10k	0.452 (0.182 – 0.659)	0.398 (0.123 – 0.624)	0.382 (0.114 – 0.606)
**Florida Panhandle**	South	85,487	45–100k	0.694 (0.434 – 0.869)	0.679 (0.425 – 0.848)	0.634 (0.385 – 0.804)
**Ft Leavenworth**	Midwest	15,911	10–18k	0.264 (-0.043 – 0.524)	0.145 (-0.163 – 0.438)	0.270 (-0.026 – 0.530)
**Garden State**	Northeast	25,247	18–45k	0.559 (0.328 – 0.718)	0.542 (0.315 – 0.712)	0.216 (-0.048 – 0.469)
**Great Lakes**	Midwest	48,171	45–100k	0.218 (0.191 – 0.402)	0.218 (0.191 – 0.402)	NA
**Gulf Coast**	South	34,988	18–45k	0.401 (0.087 – 0.63)	0.222 (-0.137 – 0.485)	0.358 (0.045 – 0.607)
**Hill AFB**	West	19,454	18–45k	0.249 (-0.036 – 0.494)	0.138 (-0.167 – 0.415)	0.164 (-0.103 – 0.408)
**Holloman AFB**	West	9,751	0–10k	0.287 (0.019 – 0.517)	0.229 (-0.030 – 0.464)	0.256 (-0.013 – 0.478)
**Jacksonville**	South	59,334	45–100k	0.751 (0.545 – 0.88)	0.672 (0.432 – 0.828)	0.716 (0.507 – 0.851)
**Kirtland AFB**	West	9,927	0–10k	0.443 (0.201 – 0.62)	0.334 (0.108 – 0.509)	0.380 (0.129 – 0.594)
**MacDill AFB**	South	18,517	18–45k	0.656 (0.414 – 0.817)	0.659 (0.427 – 0.808)	0.463 (0.182 – 0.680)
**McConnell AFB**	Midwest	8,715	0–10k	0.150 (-0.129 – 0.422)	0.277 (0.027 – 0.528)	-0.115 (-0.421 – 0.224)
**National Capital Region**	South	264,415	100–290k	0.686 (0.477 – 0.828)	0.648 (0.428 – 0.800)	0.695 (0.487 – 0.846)
**New England**	Northeast	35,177	18–45k	0.460 (0.219 – 0.65)	0.405 (0.148 – 0.617)	0.371 (0.134 – 0.577)
**North Carolina, Central**	South	125,861	100–290k	0.787 (0.647 – 0.862)	0.770 (0.599 – 0.863)	0.674 (0.494 – 0.791)
**North Carolina, Coastal**	South	89,929	45–100k	0.462 (0.185 – 0.676)	0.408 (0.114 – 0.638)	0.496 (0.258 – 0.688)
**Offutt AFB**	Midwest	18,591	18–45k	0.268 (0.058 – 0.456)	0.143 (-0.037 – 0.305)	0.183 (-0.014 – 0.374)
**Patrick SFB**	South	10,490	10–18k	0.582 (0.324 – 0.768)	0.554 (0.282 – 0.754)	0.445 (0.155 – 0.660)
**Puget Sound**	West	155,589	100–290k	0.839 (0.694 – 0.916)	0.744 (0.549 – 0.859)	0.824 (0.682 – 0.902)
**Scott AFB**	Midwest	16,987	10–18k	0.517 (0.293 – 0.691)	0.319 (0.069 – 0.535)	0.380 (0.119 – 0.598)
**South Florida**	South	7,491	0–10k	0.514 (0.304 – 0.662)	0.514 (0.304 – 0.662)	NA
**Tidewater**	South	195,716	100–290k	0.696 (0.504 – 0.82)	0.646 (0.432 – 0.792)	0.739 (0.581 – 0.840)
**Upstate New York**	Northeast	28,725	18–45k	0.049 (-0.226 – 0.313)	0.004 (-0.256 – 0.256)	0.089 (-0.206 – 0.360)
**Vance AFB**	South	3,430	0–10k	0.240 (-0.040 – 0.499)	0.236 (-0.059 – 0.505)	0.213 (0.010 – 0.393)
**West Point**	Northeast	15,125	10–18k	-0.095 (-0.378 – 0.175)	-0.108 (-0.381 – 0.173)	-0.022 (-0.304 – 0.249)

**Whiteman AFB**	Midwest	10,264	10–18k	0.385 (0.150 – 0.581)	0.301 (0.060 – 0.522)	0.280 (0.009 – 0.516)
**Wright-Patterson AFB**	Midwest	35,493	18–45k	0.684 (0.489 – 0.804)	0.580 (0.331 – 0.750)	0.663 (0.476 – 0.786)

Note: AFB = Air Force Base, MHS = Military Health System, AD SM = Active-Duty Service Member, *rs* = Spearman correlation coefficient, CI = confidence interval, NA = Not Applicable

Variable	Description	Source
**mhs_covid_cases**	MHS COVID-19 cases defined by positive laboratory test (SARS-CoV-2 PCR or antigen test)	DoW ESSENCE and DMSS
**ad_covid_cases**	AD SM COVID-19 cases defined by positive laboratory test (SARS-CoV-2 PCR or antigen test)	DoW ESSENCE and DMSS
**non_ad_covid_cases**	Subset of MHS COVID-19 cases who are not AD SMs	DoW ESSENCE and DMSS
**date**	Date of SARS-CoV-2 specimen collection	DoW ESSENCE and DMSS
**site**	A unique identifier for wastewater treatment plants. This is an arbitrary integer used to provide a unique, but anonymous identifier for a wastewater treatment plant. This identifier is consistent over time, such that the same plant retains the same ID regardless of the addition or subtraction of other plants from the data set.	NWSS
**county_fips**	5-digit numeric FIPS codes of all counties and county equivalents served by this sampling site (i.e., served by this wastewater treatment plant or, if 'sample_location' is "upstream", then by this upstream location). Note that most jurisdictions are covered by counties, but some are covered by county equivalents, such as independent cities, parishes, or census areas.	NWSS
**sample_collect_date**	Sample collection date. ([yyyy]-[mm]-[dd])	NWSS
**pcr_target_avg_conc**	SARS-COV-2 wastewater concentration. Concentration of the PCR target back-calculated to unconcentrated sample basis; value is "0" if no amplification occurred, using the definition of amplification described in 'ntc_amplify'; otherwise, the estimated concentration was entered; does not adjust for matrix recovery efficiency. Only the technical or biological replicate concentrations for the same pcr_gene_target are averaged. In units of copies/L.	NWSS

Supplementary Table 2. Study Data Dictionary.
